# Giant Cell Arteritis Masquerading As Migraine: A Case Report

**DOI:** 10.7759/cureus.44107

**Published:** 2023-08-25

**Authors:** Sujata Devi, Anil Dash, Suvendu Purkait, Biswajit Sahoo

**Affiliations:** 1 Internal Medicine, All India Institute of Medical Sciences, Bhubaneswar, Bhubaneswar, IND; 2 Pathology and Laboratory Medicine, All India Institute of Medical Sciences, Bhubaneswar, Bhubaneswar, IND; 3 Radiology, All India Institute of Medical Sciences, Bhubaneswar, Bhubaneswar, IND

**Keywords:** migraine headaches, giant cell arteritis with polymyalgia rheumatica, temporal arteritis, migraine, giant cell arteritis

## Abstract

Giant cell arteritis, or temporal arteritis, is a chronic granulomatous vasculitis that affects large- and medium-sized arteries. An elderly male of 61 years presenting with chronic headaches for the past one year had been misdiagnosed as having migraine because of the similarity in symptoms. General examination revealed the presence of bilateral large, tortuous temporal arteries without any scalp tenderness, diminished arterial pulsations, or skin changes over the dilated arteries. A temporal artery biopsy revealed giant cell arteritis and was treated with steroids. This case report highlights the importance of considering secondary headaches, especially giant cell arteritis, in the differential diagnosis of new-onset headaches or worsening headaches in the elderly.

## Introduction

Giant cell arteritis (GCA) is an autoimmune inflammatory condition that affects large- and medium-sized arteries. The age of onset is usually over 50 years and females are more affected than males in a ratio of 3:1 [1]. Its clinical manifestations vary and include headache, scalp tenderness, vision loss, and jaw claudication [2]. It is important to differentiate headaches secondary to GCA from migraines so that early treatment can be started for the former and devastating complications like permanent visual loss avoided.

Here, we report the case of an elderly male who presented with features mimicking a migraine headache for the past year before being diagnosed correctly with GCA.

## Case presentation

A 61-year-old male presented to the outpatient department with a history of headaches for one year. The headache was bilateral, dull aching, moderate-severe in intensity, occurring over the temples and in the retro-orbital region, and usually lasted 10-12 hours. It was relieved with non-steroidal anti-inflammatory drugs (NSAIDs) and occurred almost daily. The patient reported occasional episodes of nausea during the headache attacks. The headache was not associated with photophobia or phonophobia. There were no specific triggers for the onset of headache, and no history of aura could be elicited. The headache episodes were not associated with autonomic phenomena such as lacrimation or nasal congestion.

Along with the onset of headaches, the patient also complained of generalized weakness and malaise with a loss of appetite for one year. The patient also complained of pain and stiffness in the upper back, especially during the morning, which was relieved with movement. There was no history of fever, altered sensorium, difficulty in chewing food, or visual abnormalities. Before presenting to our outpatient department, the patient had been diagnosed with migraine and started on amitriptyline, propranolol, and flunarizine as a preventive treatment for the same for the last six months without relief.

General examination revealed the presence of bilateral large and tortuous temporal arteries without any scalp tenderness, diminished arterial pulsations, or skin changes over the dilated arteries. The rest of the general and systemic examinations were within normal limits. Fundoscopy was within normal limits and no refractive error was found.

Basic laboratory investigations at the time of admission were found to be normal. The erythrocyte sedimentation rate (ESR) level was elevated (120 mm/hour) while the C-reactive protein (CRP) level was also elevated (12 mg/L). Non-contrast computed tomography (NCCT) of the brain was normal. Greyscale ultrasound with color Doppler of bilateral temporal arteries revealed only mild atherosclerotic changes bilaterally without any evidence of inflammatory changes in the form of mural thickening or luminal stenosis.

**Figure 1:**
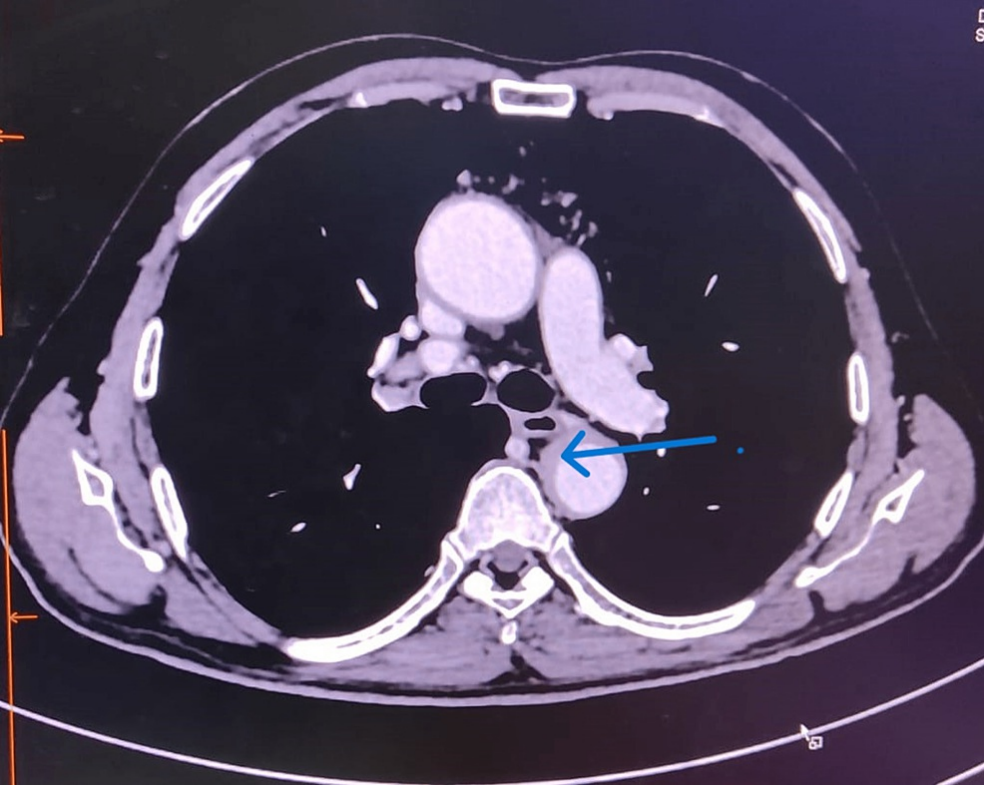
CT angiogram study of the aorta shows an asymmetric circumferential mural thickening (arrow) in the descending thoracic aorta.

Because of high clinical suspicion of GCA, the patient was advised for a CT angiogram (CTA) study of the aorta and its branches (Figure 1). The CTA revealed asymmetric circumferential mural thickening in the distal arch of the aorta and descending thoracic aorta with a maximum thickness of ~5.3 mm. Mild circumferential wall thickening was also noted in the abdominal aorta involving bilateral renal ostia, and intimal calcification was noted in the descending thoracic aorta and abdominal aorta; however, no apparent luminal narrowing was noted in the aorta or its branches. There was no evidence of aneurysm/dissection/mural thrombus or hematoma. Magnetic resonance imaging (MRI) of the brain with MR angiography revealed only chronic small vessel ischemic changes in the bilateral frontoparietal region.

**Figure 2:**
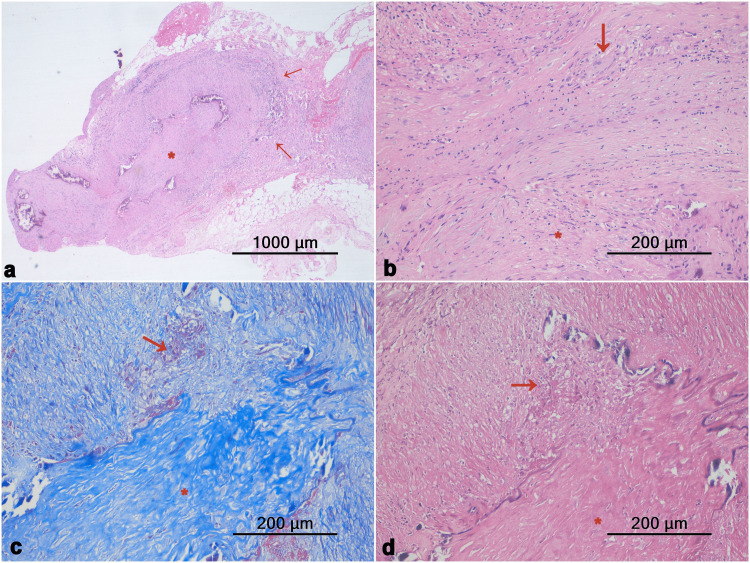
(a, b) Microscopic examination of vessel wall shows an organized fibrotic thrombus in the lumen (*) and inflammatory cell infiltration in the wall (arrow); (c, d) MT and VVG staining shows fragmentation of the internal elastic lamina (arrow) with fibrotic thrombus (*). MT: Masson's trichrome stain; VVG: Verhoeff-van Gieson stain

On review of the CTA report, which was suggestive of aortoarteritis, the patient was planned for a temporal artery biopsy. A special stain revealed fragmentation of internal elastic lamina and an impression of GCA was reported (Figure 2). The patient was diagnosed with GCA and started on steroids. He received a pulse dose of methylprednisolone 1 gm for three consecutive days followed by oral prednisolone 50 mg once daily. Along with the steroid, methotrexate 10 mg once weekly was given and a tablet of folic acid 5 mg weekly twice was added.

The patient reported a significant improvement in the headache and myalgia symptoms after receiving the pulse dose of steroids. The patient was advised to continue taking oral prednisolone 50 mg daily dose for four weeks and then plan to taper and continue tablet methotrexate.

## Discussion

In addition to the characteristic symptoms of headache, scalp tenderness, visual loss, and jaw claudication, GCA can present with non-specific systemic symptoms like fever, fatigue, or weight loss [2]. It can also present with symptoms of polymyalgia rheumatica including characteristic proximal polyarthralgia and myalgias. Atypical presentations in GCA include ischemic stroke, respiratory symptoms (sore throat, hoarseness, dry cough), dysarthria, sensorineural hearing loss, throat pain, tongue pain, macroglossia, lingual infarction, pericarditis, mesenteric ischemia [3].

Aortic involvement in GCA is frequently missed due to the paucity of clinical manifestations. In a study by Prieto-Gonzalez et al., CTA-defined vasculitis of the aorta was detected in 65% of GCA patients at the time of diagnosis [4]. GCA patients with aortitis at the time of diagnosis are at an increased risk for aortic aneurysms, most notably in the ascending thoracic aorta [5].

Laboratory evaluation in GCA reveals normochromic normocytic anemia, thrombocytosis, hypoalbuminemia, elevated ESR, CRP, and a raised fibrinogen.

Imaging evaluation in GCA includes a CDUS of the arteries in the head, neck, and upper extremities, especially the temporal artery. A meta-analysis by Arida et al. revealed a weighted sensitivity of 68% and specificity of 91% for CDUS compared to the temporal artery biopsy [6]. In our case as well, CDUS of the bilateral temporal artery did not reveal any inflammatory changes.

Temporal artery biopsy is used for confirmation of diagnosis but may not be positive in all cases given the patchy histological findings. Histological findings include transmural inflammatory infiltrate, giant cells, laminar necrosis, medial attenuation, fragmentation of internal elastic lamina, and adventitial fibrosis.

Treatment options in GCA include glucocorticoids, tocilizumab, methotrexate, azathioprine, and abatacept.

## Conclusions

This case report highlights the importance of including GCA in the differential diagnosis of chronic headaches, especially in the elderly population. Early diagnosis of this entity and institution of appropriate treatment may prevent the dreaded ocular complications associated with the disease and improve the quality of life.
